# Compound refractive lenses as prefocusing optics for X-ray FEL radiation

**DOI:** 10.1107/S1600577516001636

**Published:** 2016-02-17

**Authors:** Philip Heimann, Michael MacDonald, Bob Nagler, Hae Ja Lee, Eric Galtier, Brice Arnold, Zhou Xing

**Affiliations:** a Linac Coherent Light Source, SLAC National Accelerator Laboratory, 2575 Sand Hill Road, Menlo Park, CA 94025, USA; b Applied Physics Program, University of Michigan, 500 South State Street, Ann Arbor, MI 48109, USA

**Keywords:** compound refractive lens, X-ray free-electron laser, X-ray optics

## Abstract

The performance of X-ray free-electron laser beamlines may be limited by the angular aperture. Compound refractive lenses (CRLs) can be employed to prefocus the X-ray beam, thereby increasing the beamline transmission. A prefocusing CRL was implemented in the X-ray transport of the Matter under Extreme Conditions Instrument at the Linac Coherent Light Source. A significant improvement in the beamline transmission was calculated over the 3–10 keV photon energy range. At 5 keV, the relative X-ray intensity was measured and a factor of four increase was seen in the beamline transmission. The X-ray focus was also determined by the ablation imprint method.

## Abstract 2

A prefocusing compound refractive lens was implemented for the Matter under Extreme Conditions Instrument at the Linac Coherent Light Source. A significant improvement in the beamline transmission was calculated and observed at 5 keV.

## 1. Introduction   

X-ray free-electron laser (FEL) experiments are performed over a range of photon energies. The Linac Coherent Light Source (LCLS) provides fundamental radiation at photon energies from 280 to 11000 eV. The divergence of X-ray FEL radiation is small, but increases approximately linearly with wavelength, λ. However, X-ray FEL beamlines are rather long compared with those at synchrotron radiation facilities. For the LCLS instruments in the Far Experimental Hall, the X-ray beam is transported ∼400 m. At the LCLS the focusing elements, compound refractive lenses (CRLs) or Kirkpatrick–Baez mirrors, are located in the instrument hutches, far from the source. Consequently, the angular aperture is limited, which may reduce the beamline transmission especially at the intermediate X-ray photon energies, 3–6 keV.

To improve the beamline transmission, it is advantageous to introduce a CRL at a distance between the X-ray FEL source and the X-ray instrument as a prefocusing optic. CRLs are well suited for this purpose. They do not alter the central beam direction, and a single lens set focuses in both the horizontal and vertical directions. To produce a weakly converging beam, these prefocusing lenses have large radii and consequently a considerable aperture. The X-ray beam dimensions can thus be matched to the aperture of the focusing optics in the X-ray instrument and the transmission increased.

Prefocusing was implemented at the LCLS Matter under Extreme Conditions (MEC) Instrument (Nagler *et al.*, 2015 ▸). This X-ray instrument hosts investigations of plasmas and high-pressure phase transitions. The instrument includes high-intensity and high-energy laser systems, which generate high-temperature and high-pressure states. CRLs are used to focus the X-rays to a spot size smaller than that of the optical beam. In the case of X-ray heating experiments, smaller focal diameters allow high electron temperatures of 104–106 K to be accessed (Fletcher *et al.*, 2013 ▸). Prefocusing CRLs are also included in the design of the European XFEL Materials Imaging and Dynamics and High Energy Density Science Instruments (Madsen *et al.*, 2013 ▸; Nakatsutusmi *et al.*, 2014 ▸).

Here we present modeling of the MEC beamline transmission with and without prefocusing CRLs. Commissioning of the prefocusing CRLs was performed and the transmission measured at 5 keV. Lastly, the X-ray focusing was characterized with the ablation imprint method.

## 2. Modeling   

CRLs consist of two concave parabolic surfaces in a low-*Z* material such as beryllium or aluminium (Snigirev *et al.*, 1996 ▸; Lengeler *et al.*, 1999 ▸). They focus through the real part of the index of refraction. They are easily aligned by centering the lens stack on the X-ray beam. CRLs have a nominal aperture, given by a diameter
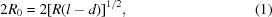
where *R* is the radius of curvature at the apex, *l* is the thickness and *d* is the distance between the apices of the parabolic surfaces. In addition, the effective aperture of CRLs is limited by X-ray absorption (Lengeler *et al.*, 1999 ▸).

**Figure 1:**
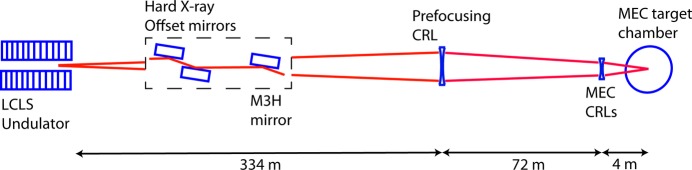
Schematic diagram of the LCLS MEC X-ray beamline.

As shown in Fig. 1 ▸, the MEC X-ray transport includes two hard X-ray offset mirrors, which satisfy radiation safety requirements, and a M3H mirror, which deflects the X-ray beam from the other instruments in the Far Experimental Hall (Nagler *et al.*, 2015 ▸). The offset and M3H mirrors have an incidence angle of 1.325 mrad and a length of 450 mm. Their figure error specification was 2 nm and 0.25 µrad r.m.s. (Soufli *et al.*, 2009 ▸; McCarville *et al.*, 2008 ▸). The initial construction of the MEC instrument included CRLs for X-ray focusing within the hutch. The MEC CRL assembly holds three CRLs, which can be alternately translated into the X-ray beam. An inventory of refractive lenses is available with radii from 50 to 5000 µm. The assembly can be translated such that the distance from the CRLs to the target chamber center can be varied from 3.9 to 4.4 m. The three plane mirrors limit the horizontal aperture, but without prefocusing the CRLs at the instrument provide a smaller limit to the angular aperture of the beamline.

**Figure 2:**
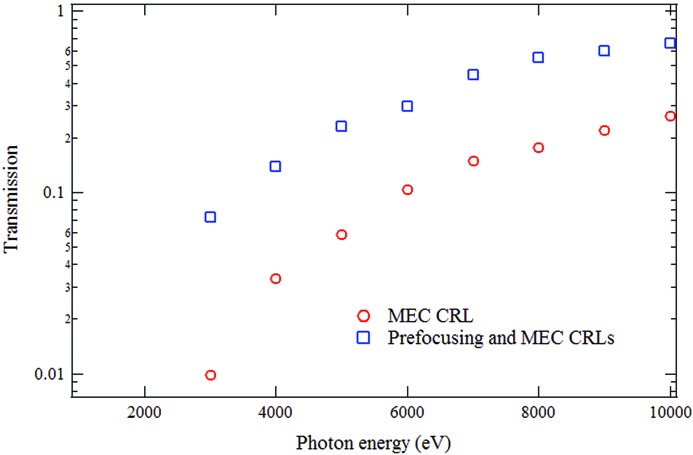
Calculated transmission of the MEC CRL alone (red circles) and with an additional CRL acting as prefocusing optic (blue squares).

Assuming a Gaussian beam profile, the transmission *T* of a refractive lens is given by
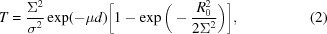


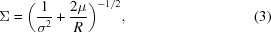
where μ is the sum of the atomic photoabsorption and inelastic scattering cross sections and σ is the standard deviation of the Gaussian function. In actuality, because of the finite aperture and figure imperfections of the hard X-ray offset and M3H mirrors, the unfocused X-ray beam at MEC has a multiple peak structure in the horizontal direction and a nearly Gaussian shape in the vertical direction. At the sample and for the example of 5 keV with a prefocusing CRL, the transmission corresponds to 9 × 10^11^ photons per pulse and an intensity of 2 × 10^17^ W cm^−2^ assuming a 3 µm focus. Fig. 2 ▸ shows the calculated transmission of the CRLs at the MEC instrument. These computations use the Gaussian approximation for the X-ray beam profile and equations (2) and (3). The observed LCLS X-ray divergence is taken into account as well as the source location within the undulator, which varies with photon energy (Turner *et al.*, 2015 ▸, 2011 ▸). The apertures of the offset and M3H mirrors have been included. For reference at 5 keV photon energy, the calculated beam size is 1.0 mm (full width at half-maximum) at the M3H mirror and 1.9 mm (FWHM) at the prefocusing CRL. At the MEC CRL and at 5 keV, the beam dimension is 2.2 mm (FWHM) without the prefocusing CRL and 0.4 mm (FWHM) with the pre­focusing CRL. After the offset and M3H mirrors, these values are appropriate for the vertical direction.

Two configurations are considered. In case 1 (red circles), the CRLs in the MEC instrument are the sole focusing element. In case 2 (blue squares), a prefocusing CRL has been added 72 m upstream of the MEC CRLs. The additional CRL weakly focuses the X-rays reducing the beam diameter at the MEC CRLs. The prefocusing CRL has a large radius and hence a large aperture. In this case with the prefocusing CRL, the horizontal aperture of the mirrors, in particular the M3H mirror, provides an upper limit on the beamline transmission.

Table 1 ▸ lists the CRL options used in the transmission calculations, with and without a prefocusing CRL and at various photon energies. For all lenses the material is beryllium. *l* is taken to be 1 mm, while *d* is assumed to be 60 µm for lenses with *R* ≥ 2000 µm and 30 µm for the lenses with *R* < 2000 µm. Fig. 2 ▸ shows that the combined transmission of prefocusing and MEC CRLs is predicted to improve dramatically. At 5 keV, the calculated transmission increases by a factor of 3.9.

## 3. Intensity measurements   

A manipulator was installed for prefocusing CRLs in the X-ray transport tunnel between the LCLS Near and Far Experimental Halls. In an accident condition, if the X-ray FEL were tuned to an incorrect photon energy, the prefocusing CRL could focus on and damage the radiation stoppers. Initially for reasons of simplicity, the prefocusing CRLs were installed upstream but close to the MEC radiation stoppers. Later, the positions of the prefocusing CRLs and radiation stoppers were interchanged.

The relative X-ray intensity was measured with an LCLS intensity position monitor (Feng *et al.*, 2011 ▸) located downstream of the MEC CRLs. In this diagnostic, four photodiodes measure X-rays backscattered from an Si_3_N_4_ foil. The parameters of the prefocusing CRL are listed in Table 2 ▸. This lens produced a virtual focus 18 m downstream of the MEC CRLs. In the intensity measurements, the prefocusing and MEC CRL parameters are shown in Table 1 ▸ for 5 keV and are the same as those used in the transmission calculation at that photon energy. In combination with the prefocusing CRL, the CRL in the instrument requires fewer or larger radii lenses. Whereas these intensity measurements were performed at 5 keV, prefocusing CRLs are available over the 3–10 keV photon energy range.

**Figure 3:**
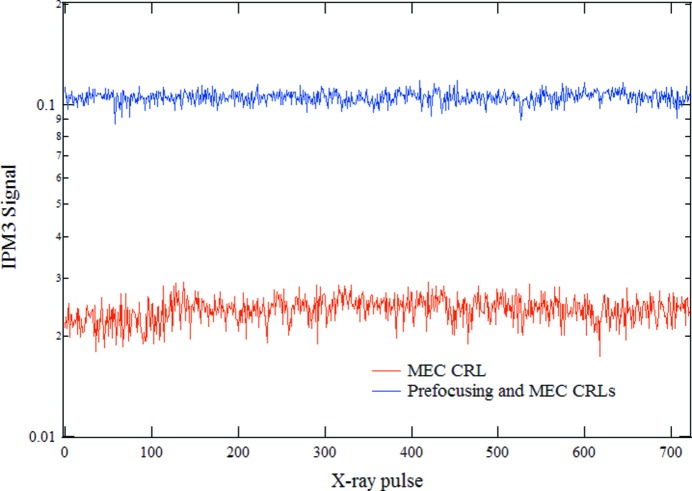
The measured relative X-ray intensity at 5 keV with and without the prefocusing CRL.

Fig. 3 ▸ shows the relative X-ray intensity at 5 keV photon energy on the intensity position monitor downstream of the MEC CRLs with and without the prefocusing CRL and using the appropriate MEC CRL in each case. The intensities were saved shot by shot. At 5 keV, an average improvement of 4.4 was observed. This measured result and the calculated value of 3.9 are in satisfactory agreement. A project has been started to replace the hard X-ray offset and M3H mirrors with longer mirrors. The higher measured intensity confirms the expected performance of the prefocusing CRL.

## 4. Focus measurements   

Ablation imprints have been shown to be an effective way to characterize the X-ray focal spot of X-ray FEL radiation (Chalupský *et al.*, 2010 ▸, 2007 ▸). The imprint targets used in this study consisted of 50 nm of gold deposited on a fused silica substrate. The thin layer of gold has a sharp threshold fluence at which it ablates. The spatial profile of the X-ray beam is measured by varying the pulse energy using various attenuators. Here, it is assumed that the X-ray beam profile can be approximated by two Gaussian functions. In actuality, the focused X-rays at the LCLS MEC instrument are observed to deviate from a Gaussian shape in part because of the aperturing and figure errors of the hard X-ray offset and M3H mirrors. Nevertheless, this analysis with Gaussian functions can represent the focal area.

Beginning with a single Gaussian profile, the threshold fluence for ablation is given by
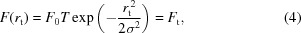
where *T* is the transmission through the attenuators, *F*_0_ is the maximum fluence and *r*_t_ is the radius at the threshold fluence. In this analysis we measure the total area, *A*, of the focal spot using a program which employed an edge detection algorithm to accurately measure the area of imprint patterns with arbitrary shapes. The area of a Gaussian spot is given by
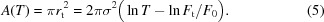
The threshold fluence is given by the *x*-intercept when the area of the ablated spot goes to zero. In our analysis we observed two linear regions in the data, implying that the X-ray beam profile is better represented by the sum of two Gaussian profiles. To model this we assume a fluence of the form
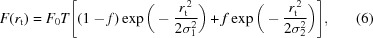
where the first Gaussian function corresponds to the main central component, the second to the wings of the focus and *f* is a weighting factor.

**Figure 4:**
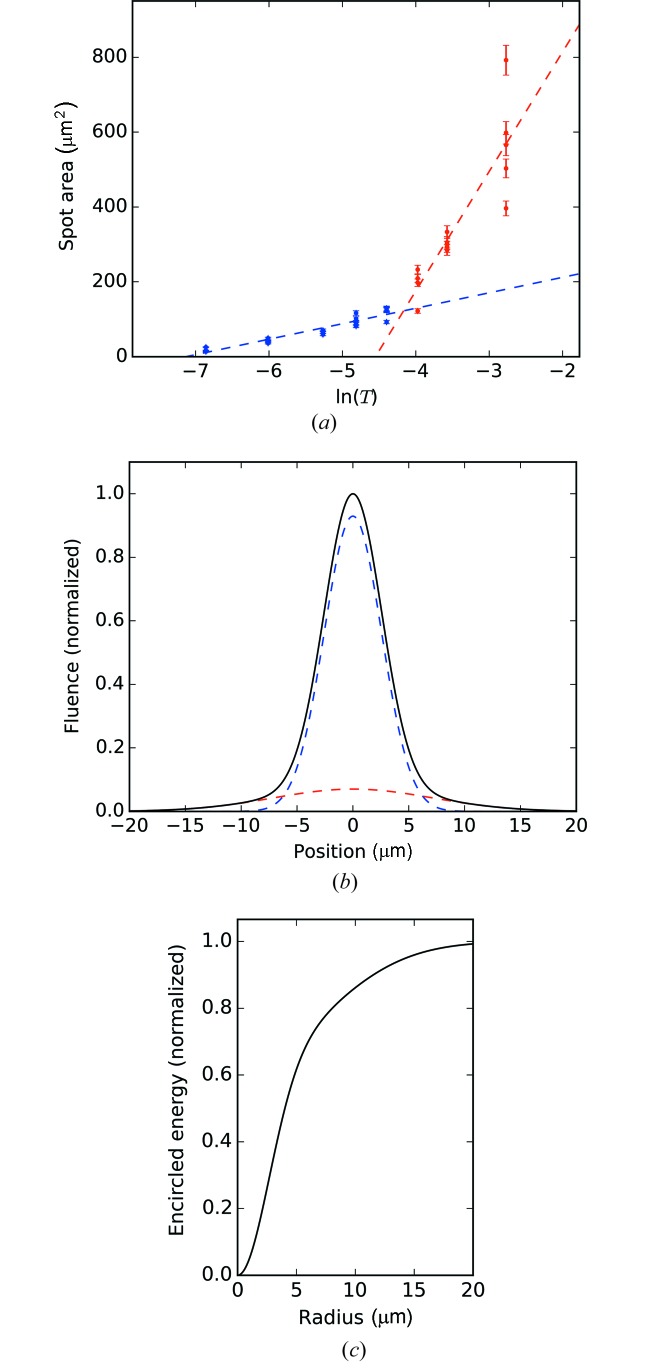
(*a*) Ablation imprint area *versus* transmission, (*b*) fit fluence profile and (*c*) encircled energy. The blue and the red curves represent the two Gaussian contributions to the X-ray focus and the solid black fluence curve is the sum.

The energy encircled within a defined radius is a useful quantity for the beam because it gives the percentage of photons within the given radius. This is calculated by integrating the fluence:
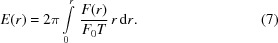
Fig. 4 ▸ shows the ablation imprint analysis for the focusing configuration including the prefocusing and MEC CRLs. The ablation area is plotted as a function of 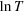. The two linear regions represent contributions from the high intensity, narrow Gaussian (blue), and the lower intensity, broad Gaussian (red). The error of the area measurement was determined to be 5% by adjusting the edge detection parameters in the analysis script and comparing the results with the microscope images. These results show that the fluence of the X-ray beam can be approximated as the sum of two Gaussian profiles with FWHMs of 6 and 17 µm and *f* = 0.07. The encircled energy analysis shows that 50% of the energy is contained within an 8 µm diameter. It is noted that there is a non-circular aspect to the focus not captured in Fig. 4 ▸. Compared with this measured result, the calculated focus including the diffraction limit and chromatic aberration is 3 µm FWHM. Ablation imprints were performed at only three translations of the MEC CRLs, and a smaller focus at a different CRL translation cannot be excluded. In addition, the aperturing and imperfections of the hard X-ray offset and M3H mirrors and the imperfections of the CRLs will contribute to the measured X-ray focus. For comparison without a prefocusing CRL, the calculated focal width is 2 µm at 5 keV, and a focal dimension of 2 µm was previously measured at 7.6 keV (Nagler *et al.*, 2015 ▸). In general, prefocusing CRLs need not compromise the focal size because the X-ray beam dimension may be matched to the effective diameter of the CRL in the hutch, which determines the focal size in the diffraction limit.

## 5. Conclusion   

For X-ray FEL beamlines, the angular aperture is important for the transmission, especially at lower photon energies. A prefocusing CRL can match the X-ray beam dimensions to the aperture of a CRL or Kirkpatrick–Baez mirrors in an X-ray FEL instrument. A prefocusing CRL was installed in the LCLS MEC beamline. At 5 keV photon energy an improvement by a factor of four was observed in the X-ray transmission. The X-ray focus was characterized showing that 50% of the intensity was contained within an 8 µm diameter.

## Figures and Tables

**Table 1:** CRL options for the two configurations *N* is number of lenses and *R* is the radius (µm) of the lens.

	Photon energy (keV)							
	3	4	5	6	7	8	9	10
Case 1								
MEC CRL								
*N* × *R*	3 × 1000,	3 × 500	2 × 300,	6 × 500,	9 × 500	7 × 300	9 × 300	11 × 300
	1 × 3000		1 × 1000,	1 × 1000				
			2 × 1500					
Case 2								
Prefocusing CRL								
*N* × *R*	1 × 5000	1 × 3000	1 × 2000	2 × 3000	2 × 2000	2 × 1500	1 × 1000,	2 × 1000
							1 × 1500	
MEC CRL								
*N* × *R*	2 × 1500	4 × 1000	3 × 500,	5 × 500,	7 × 500	5 × 300,	7 × 300	9 × 300
			1 × 1000	1 × 1000		1 × 1000		

**Table 2:** Parameters of the prefocusing CRL used in the intensity measurements at 5 keV

Material	*R* (µm)	*l* (µm)	*d* (µm)	2*R*_0_ (mm)
Beryllium	2000	1000	50	2.8
